# Dupla Via de Saída de Ventrículo Direito com Comunicação Interventricular não Relacionada e Estenose Pulmonar, em Evolução Natural, em Mulher com 36 Anos

**DOI:** 10.36660/abc.20200971

**Published:** 2021-04-08

**Authors:** Edmar Atik

**Affiliations:** 1 Universidade de São Paulo Instituto do Coração Hospital das Clínicas São PauloSP Brasil Instituto do Coração do Hospital das Clínicas da Faculdade de Medicina da Universidade de São Paulo, São Paulo, SP – Brasil

**Keywords:** Dupla Via de Ventrículo Direito, Cardiopatias Congênitas/cirurgia, Comunicação Interventricular, Diagnóstico por Imagem, Estenose da Valva Pulmonar, Adulto

## Introdução

O defeito congênito caracterizado por saída de ambas grandes artérias do ventrículo direito (VD), de forma completa ou mesmo em cavalgamento superior a 50% de uma das artérias sobre o septo interventricular, apresenta relações variáveis dessas artérias com a comunicação interventricular (CIV), assim como associação com outras variáveis anátomo-funcionais.^1^

No defeito mais comum associado, a CIV isolada, em posição subaórtica ou na não relacionada às grandes artérias, o quadro funcional se expressa com predomínio da sobrecarga de volume do coração como um todo, incrementada pela sobrecarga de pressão, causando insuficiência cardíaca precoce.

Em defeitos associados como coartação da aorta, estenose mitral e ainda quando a CIV se mostra restritiva, há acentuação do quadro congestivo pulmonar. Associação com defeito do septo atrioventricular, anomalias de posição cardíaca e de isomerismos atriais, também reforçam este quadro.

Na associação da CIV com estenose pulmonar infundíbulo-valvar, surge outro tipo de complicação da dinâmica cardiovascular, responsável pelo aparecimento de quadros variados de hipóxia. A cianose é progressivamente mais intensa na dependência da acentuação da estenose pulmonar, quadro similar àquele apresentado na tetralogia de Fallot. O mesmo ocorre em pacientes submetidos à bandagem pulmonar prévia.

Na CIV subpulmonar (tipo *Taussig-Bing*), decorrente do resultante hiperfluxo pulmonar com sobrecarga de volume das cavidades esquerdas, expressa-se daí quadro de insuficiência cardíaca congestiva com pletora venocapilar pulmonar. A hipoxemia em geral é discreta nessa condição, e se acentua quando a comunicação interatrial se mostra restritiva. A hipóxia se mostra mais intensa em associação com estenose pulmonar. A exteriorização clínica precoce nos primeiros dias de vida é similar à encontrada na transposição das grandes artérias com CIV.

Em linhas gerais, variações clínicas dependem da intensidade dos defeitos obstrutivos, do tamanho das comunicações intercavitárias e de defeitos associados, que no conjunto incrementam a dinâmica cardiovascular.

Por vezes, na associação de estenose pulmonar e CIV, pode haver balanceamento dos fluxos, sistêmico e pulmonar, de tal modo que o paciente evolui até a idade adulta sem manifestações, mas com possíveis futuras complicações, dada a sobrecarga de pressão imposta ao VD.

Nesse diapasão, se torna discutível a conduta expectante clínica que se adota em pacientes que estejam evoluindo favoravelmente em idades mais precoces, quando crianças ou jovens.

Esta seria a razão principal da discussão e apresentação do caso a seguir.

## Descrição do Caso

### Dados clínicos:

Paciente com 36 anos de idade evolui com palpitações há 8 anos por extrassístoles ventriculares e supraventriculares, mesmo com o uso de propafenona. Refere boa tolerância a esforços físicos usuais e usa levotiroxina por hipotireoidismo. Endocardite infecciosa foi tratada com 17 anos de idade. A família rejeitou a ideia de intervenção cirúrgica na primeira década da vida, em vista de que nesta época a paciente se encontrava em bom estado geral e sem sintomas.

### Exame físico:

bom estado geral, eupneica, acianótica, pulsos normais nos 4 membros. Peso: 55 Kg, Alt.: 165 cm, PAMSD: 100x65 mmHg, FC: 79 bpm, Sat.O_2_= 96%.

### Precórdio:

*ictus cordis* não palpado, sem impulsões sistólicas na borda external esquerda. Bulhas cardíacas hiperfonéticas, sopro sistólico moderado em borda external esquerda alta, sem frêmito, 3/6+. Fígado não palpado e pulmões limpos.

## Exames Complementares

### Eletrocardiograma:

**Figura 1:**
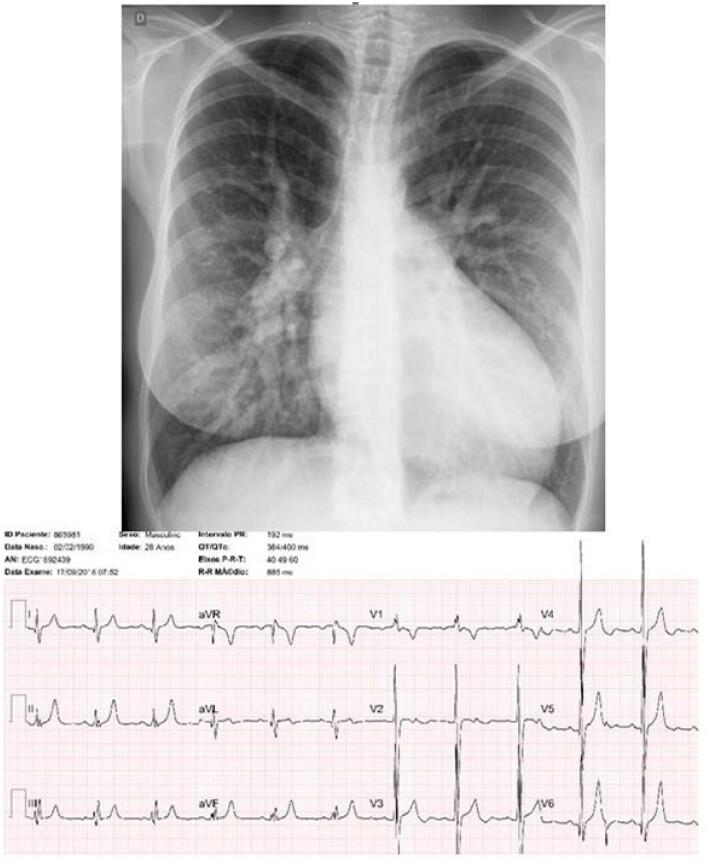
Radiografia de tórax mostra o aumento discreto a moderado da área cardíaca a custa do arco ventricular esquerdo alongado e arredondado (ICT=0,57). Trama vascular pulmonar aumentada estando bem saliente no hilo à direita com arco médio abaulado. Eletrocardiograma salienta a sobrecarga diastólica de ambos os ventrículos.

Ritmo sinusal, com morfologia “rs” em V1 estando a onda “r” espessada e entalhada (AQRS= +60º). Havia sobrecarga ventricular direita tipo diastólica com onda T negativa em V1, e presença de potenciais esquerdos com complexo qRs em V6, sendo altas as ondas R de V4 a V6. Não havia alterações da repolarização ventricular (AT= +60º), onda P normal (AP= +50º) (Figura 1).

### Radiografia de tórax:

Aumento discreto a moderado da área cardíaca a custa do arco ventricular esquerdo, longo e arredondado (ICT=0,57). Trama vascular pulmonar aumentada estando mais saliente no hilo à direita, com arco médio abaulado. Arco aórtico normal (Figura 1).

### Ecocardiograma:

**Figura 2:**
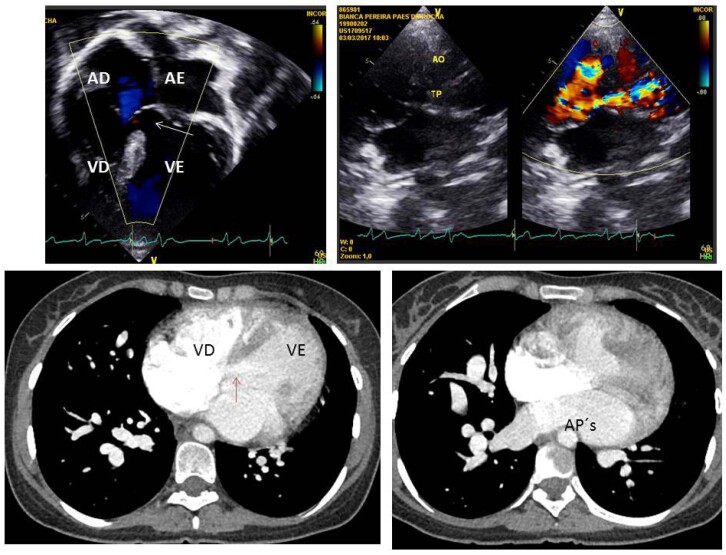
Ecocardiograma mostra em corte de 4 câmaras a grande comunicação interventricular (seta) de via de entrada e em corte subcostal os dois grandes vasos emergindo do ventrículo direito estando a aorta à direita da pulmonar. A obstrução pulmonar se inicia na região infundibular. A tomografia cardíaca salienta as cavidades ventriculares e as artérias pulmonares dilatadas além da comunicação interventricular (seta). AD: átrio direito; AE: átrio esquerdo; VD: ventrículo direito; VE: ventrículo esquerdo; Ao: aorta; TP: tronco pulmonar; AP´s: artérias pulmonares.

Conexão atrioventricular concordante e dupla via de saída de VD com aorta anterior e à direita. A veia cava inferior era dilatada com 21 mm, com contraste espontâneo. A CIV de via de entrada com extensão para a via de saída era ampla e não relacionada, media 26 mm, com fluxo bidirecional, preferente da esquerda à direita e sem restrição, e sem gradiente de pressão interventricular. Os átrios eram aumentados, especialmente à esquerda (AE=51 mm). VD hipertrófico e dilatado com função sistólica preservada. Na via de saída havia estenose infundibular e também a nível da valva pulmonar, com gradiente sistólico de 85 mmHg. O ventrículo esquerdo (VE) era hipertrófico e dilatado (67 mm) com função normal. A aorta tinha 35 mm e as artérias pulmonares confluentes, a direita com 28 mm e a esquerda com 24 mm. A valva tricúspide tinha 30 mm e a valva mitral 25 mm (Figura 2).

### Angiotomografia:

O diagnóstico foi confirmado com medidas semelhantes às do ecocardiograma, estando aumentados o átrio esquerdo e as duas cavidades ventriculares. A função biventricular era normal. A artéria pulmonar era posterior à esquerda e a aorta à direita e anterior (Figura 2).

### Holter:

Extrassístoles ventriculares (3% do total de batimentos), sem taquicardias supraventriculares ou ventriculares. A frequência cardíaca variou de 51 a 116 bpm, com média de 76 bpm.

### Ergoespirometria:

Consumo máximo de oxigênio ajustado para o peso corporal de 22,3 ml/kg/min. A pressão arterial em repouso era de 100x60 mmHg com 75 bpm e no esforço máximo de 130x60 mmHg, com 155 bpm.

### Diagnóstico clínico:

Dupla via de saída de VD com a aorta anterior e à direita, com grande comunicação interventricular de via de entrada, não relacionada, e estenose pulmonar infundíbulo-valvar, em evolução natural na idade adulta.

## Características Clínicas

### A) Raciocínio Clínico:

Havia elementos clínicos de orientação diagnóstica da cardiopatia congênita, com malposição arterial pela hiperfonese de bulhas cardíacas e da estenose pulmonar em presença de sopro sistólico de ejeção na área pulmonar, com irradiação à borda external esquerda. A sobrecarga diastólica de VD no eletrocardiograma com potenciais nítidos de VE expressam a presença de dois ventrículos bem formados e daí se invoca presença de CIV associada. O defeito obstrutivo pulmonar contrabalança o da CIV, de tal maneira que a paciente permaneceu sem cianose, com fluxo preferencial da esquerda para a direita e sem sintomas. O diagnóstico clínico elaborado foi bem estabelecido pela ecocardiografia e tomografia.

### B) Diagnóstico diferencial:

Este quadro geral pode ser encontrado em outros defeitos que se assemelham nesta dinâmica de comunicação intercavitária e estenose pulmonar, como na transposição das grandes artérias e na dupla via de entrada de VE ou VD, nas atresias das valvas atrioventriculares e na transposição corrigida das grandes artérias. Outros elementos dos exames complementares usuais os diferenciam.

### Conduta:

Em face do balanceamento dos fluxos pulmonar e sistêmico ao longo do tempo, com ausência de sinais de hipoxemia e/ou de insuficiência cardíaca e na presença de boa tolerância física, foi considerada a continuidade da conduta expectante clínica.

## Discussão

A evolução natural desta paciente até a idade adulta nos salienta elementos desfavoráveis, embora tenha se mostrado em boas condições clínicas e hemodinâmicas. São eles os caracteres adquiridos que interferem na evolução a maior prazo de tempo adiante. Correspondem ao aumento das cavidades cardíacas, por hiperfluxo pulmonar em período anterior de tempo, e pela progressão da estenose pulmonar, com hipertrofia e dilatação cardíacas. Apesar da manutenção da boa função ventricular, a paciente está sujeita ao aparecimento de outros fatores adversos como acentuação das arritmias, insuficiência cardíaca diastólica, surgimento de hipoxemia progressiva, de endocardite infecciosa, causas do provável descontrole clínico evolutivo.^1^

Por outro lado, pouco se pode oferecer neste momento, do ponto de vista cirúrgico, pois a técnica presumível como mais adequada seria a funcional de *Fontan*, contraindicada pela ausência atual de hipóxia. A técnica corretiva seria muito difícil pela presença de CIV não relacionada e com aorta anterior.^2^ Pergunta-se daí, em casos semelhantes na idade infantil, se não seria mais conveniente a tentativa da correção, naquela faixa etária, mesmo com risco cirúrgico também considerável.

Na recordação das técnicas cirúrgicas, na CIV subaórtica se realiza a tunelização com pericárdio bovino, *dacron* ou *goretex* do fluxo sanguíneo do VE para a aorta. Este defeito pode ser ampliado quando restritivo ao fluxo, na face anterior do mesmo, evitando-se assim o feixe de condução ínfero-dorsal. Em presença de estenose pulmonar, é similar a correção àquela efetuada na tetralogia de *Fallot* com ressecção do músculo infundibular, por via atrial ou por ventriculotomia direita, ademais da valvotomia pulmonar com ampliação do anel pulmonar e posterior colocação da monocúspide. Enxerto valvulado entre o VD e o tronco pulmonar pode ser necessário, quando este se situa posteriormente ou quando a artéria coronária se posiciona na via de saída ventricular, próxima ao anel pulmonar. Na impossibilidade de correção, quando o VD se mostra hipoplásico, orienta à operação cavopulmonar total como descrita no ventrículo único funcional ou anatômico. Por sua vez, na CIV subpulmonar, o VE é direcionado ao tronco pulmonar. Dessa maneira, a troca arterial e das artérias coronárias seguem as mesmas táticas preconizadas segundo a correção de *Jatene*.

A evolução pós-operatória geralmente obedece a técnica preferida e necessária conforme o tipo anatômico. Problemas mais intensos são verificados no manejo pós-operatório quando se realiza a colocação de tubos de conexão entre o VD e o tronco pulmonar, em vista de obstrução e/ou de insuficiência valvar na evolução.

Arritmias podem complicar a evolução quando em associações com isomerismos atriais, disfunção ventricular e em defeitos residuais pós-operatórios.

No motivo desta apresentação clínica, pela associação específica da estenose pulmonar e CIV não relacionada, o quadro funcional se torna dependente mais da repercussão da lesão obstrutiva. A estenose pulmonar pode diminuir a repercussão da CIV e haver um contrabalanceamento tal que os fluxos pulmonar e sistêmico se equivalem. Por isso, a paciente pode permanecer sem sobrecargas de volume e sem sintomas e evoluir adequadamente até a idade adulta, sem manifestação. No entanto, a sobrecarga sistólica do VD pela estenose pulmonar e a discreta repercussão de volume do VE podem, a maior prazo, ocasionar problemas evolutivos como insuficiência cardíaca, arritmias, que obscurecem os resultados, e colocam a vida em risco.^1^

Em presença de CIV não relacionada como no caso em exposição, a técnica idealizada por *Barbero-Marcial3* direciona o VE para a aorta com tunelização com remendos desde a CIV até a valva aórtica, e com alívio da estenose pulmonar aplicada com relativo sucesso em face de sobrevida de 86,5% após 10 anos.^4^^,^^5^ Outra técnica, como o direcionamento pela CIV à artéria pulmonar e troca arterial subsequente, também se torna viável.

Conclui-se que a conduta mais adequada nestes pacientes, mesmo com equilíbrio dos fluxos sistêmico e pulmonar, seja a da intervenção corretiva em idades mais precoces, mesmo que o paciente esteja em boa condição clínica.^5^^–^^7^
