# Supplementary material for: Dupla Via de Saída de Ventrículo Direito com Comunicação Interventricular não Relacionada e Estenose Pulmonar, em Evolução Natural, em Mulher com 36 Anos
Source: Arq Bras Cardiol. 2021 Apr 8;116(4):836–9. [Article in Portuguese] doi: 10.36660/abc.20200971 (PMC8121408; doi:10.36660/abc.20200971)
Supplement: Translation

# Translation: Double Outlet Right Ventricle with Unrelated Ventricular Septal Defect and Pulmonary Stenosis, in Natural Evolution, in a 36-Year-Old Woman

**Authors:** Edmar Atik

**Affiliations:** 1 São PauloSP Brazil Instituto do Coração do Hospital das Clínicas da Faculdade de Medicina da Universidade de São Paulo, São Paulo, SP – Brazil

**Keywords:** Double Outlet Right Ventricle, Heart Defects, Congenital/surgery, Heart Septal Defects Ventricular, Diagnostic, Imaging, Pulmonary Valve Stenosis, Adult

## Introduction

The congenital defect, characterized by the exit of both large arteries from the right ventricle (RV), completely or even when overlapping more than 50% of one of the arteries over the interventricular septum, presents variable relationships between these arteries and the ventricular septal defect (VSD), as well as association with other anatomical and functional variables.^1^

In the most common associated defect, the isolated VSD, in a subaortic position or in that unrelated to the large arteries, the functional condition is expressed with a predominance of volume overload of the heart as a whole, increased by pressure overload, causing early heart failure.

In associated defects such as coarctation of the aorta, mitral stenosis and even when the VSD is restrictive, there is an increase in the pulmonary congestive condition. Association with atrioventricular septal defect, abnormalities of cardiac position and atrial isomerism, also reinforce this picture.

In the association of VSD with infundibular and valvar pulmonary stenosis, another type of complication of cardiovascular dynamics appears, responsible for the appearance of varied hypoxia. Cyanosis is progressively more intense depending on the accentuation of pulmonary stenosis, a picture similar to that presented in the tetralogy of Fallot. The same occurs in patients submitted to previous pulmonary banding.

In subpulmonary VSD (Taussig-Bing type), accentuation of pulmonary arterial flow with volume overload of the left cavities is responsible for congestive heart failure, expressed by pulmonary venocapillary plethora. Hypoxemia is generally mild in this condition, and is accentuated when interatrial communication is restrictive. Hypoxia is more intense in association with pulmonary stenosis. Early clinical exteriorization in the first days of life is similar to that found in the transposition of the great arteries with VSD.

In general, clinical variations depend on the intensity of the obstructive defects, the size of intercavitary communications and associated defects, which together increase cardiovascular dynamics.

Sometimes, in the association of pulmonary stenosis and VSD, there may be a balance of flows, systemic and pulmonary, in such a way that the patient progresses to adulthood without manifestations, but with possible future complications, given the pressure overload imposed on the RV.

In this tuning fork, the clinical expectant conduct adopted in patients who are progressing favorably at earlier ages, as children or young people, becomes debatable.

This would be the main reason for the discussion and presentation of the following case.

## Case Description

### Clinical data:

A 36-year-old patient developed palpitations for 8 years due to ventricular and supraventricular extrasystoles, even with the use of propafenone. She reports good tolerance to usual physical exertion and uses hypothyroidism levothyroxine. Infectious endocarditis was treated at 17 years of age. The family rejected the idea of surgical intervention in the first decade of life, considering that at that time the patient was in good general condition and without symptoms.

### Physical examination:

Good general condition, eupneic, acyanotic, normal pulses in the four limbs. Weight: 55 Kg, Height: 165 cm, BP: 100x65 mmHg, CF: 79 bpm, O_2_ Sat.= 96%.

### Precordium:

Apical impulse not palpable, without systolic impulses on the left external border. Accentuated heart sounds, moderate systolic murmur on the upper left external edge, without thrill, 3/6 + intensity. No palpable liver and clear lungs.

## Complementary Exams

### Electrocardiogram:

Sinus rhythm, with “rs” morphology in V1, with the “r” wave being thickened and notched (AQRS= +60º). There was diastolic right ventricular overload with negative T wave in V1, and the presence of left potentials with qRs complex in V6, with high R waves from V4 to V6. There were no changes in ventricular repolarization (AT = + 60º), and P wave was normal (AP = + 50º) (Figure 1).

**Figure 1:** Chest X-ray shows a slight to moderate increase in the cardiac area at the expense of the elongated and rounded left ventricular arch (CTI = 0.57). Increased pulmonary vascular network being well protruding from the hilum on the right with a convex arch. Electrocardiogram highlights the diastolic overload of both ventricles.

### Chest radiography:

Mild to moderate increase in the cardiac area at the expense of the long, rounded left ventricular arch (CTI = 0.57). Increased pulmonary vascular network being more prominent in the hilum on the right, with a convex medium arch. Normal aortic arch (Figure 1).

### Echocardiogram:

Concordant atrioventricular connection and double outlet of both arteries from the RV, being the anterior aorta at right side. The inferior vena cava was dilated with 21 mm, with spontaneous contrast. The inlet VSD with extension to the outlet route was wide and unrelated, measuring 26 mm, with bidirectional flow, preferably from left to right and without restriction, and without an interventricular pressure gradient. The atria were enlarged, especially on the left (LA = 51 mm). Hypertrophic and dilated RV with preserved systolic function. In the outflow tract there was infundibular stenosis and also at the pulmonary valve level, with a systolic gradient of 85 mm Hg. The left ventricle (LV) was hypertrophic and dilated (67 mm) with normal function. The diameter of the aorta was 35 mm and the pulmonary arteries confluent, the right with 28 mm and the left with 24 mm. The tricuspid valve was 30 mm and the mitral valve was 25 mm (Figure 2).

**Figure 2:** Echocardiogram shows, in a 4-chamber view, the large interventricular communication (arrow) of the entry route and in subcostal view, the two large vessels emerging from the right ventricle with the aorta to the right of the pulmonary. Pulmonary obstruction begins in the infundibular region. Cardiac tomography highlights ventricular cavities and dilated pulmonary arteries in addition to interventricular communication (arrow). RA: right atrium; LA: left atrium; RV: right ventricle; LV: left ventricle; PT: pulmonary trunk; PA´s: pulmonary arteries.

### Angiotomography:

The diagnosis was confirmed with measurements similar to those of the echocardiogram, with the left atrium and the two ventricular cavities enlarged. The biventricular function was normal. The pulmonary artery was posterior to the left and the aorta to the right and anterior (Figure 2).

### Holter:

Ventricular extrasystoles (3% of total beats), without supraventricular or ventricular tachycardias. Heart rate ranged from 51 to 116 bpm, with an average of 76 bpm.

### Ergospirometry:

Maximum oxygen consumption adjusted for body weight of 22.3 ml/kg/min. Blood pressure at rest was 100x60 mmHg at 75 bpm and at maximum effort was 130x60 mmHg at 155 bpm.

### Clinical diagnosis:

Double outlet RV with the anterior and right aorta, with great unrelated VSD at the inlet portion, and infundibulo-valve pulmonary stenosis, in natural evolution in adulthood.

## Clinical Characteristics

### A) Clinical Reasoning:

There were clinical elements of diagnostic guidance for congenital heart disease, with arterial malposition due to accentuated heart sounds and pulmonary stenosis in the presence of systolic ejection murmur in the pulmonary area, with irradiation to the left external border. The right ventricular diastolic overload on the electrocardiogram with clear LV potentials express the presence of two well-formed ventricles and hence the presence of associated VSD. The obstructive pulmonary defect counterbalances that of VSD, in such a way that the patient remained without cyanosis, with preferential flow from left to right and without symptoms. The elaborated clinical diagnosis was well established by echocardiography and tomography.

### B) Differential diagnosis:

This general picture can be found in other defects that are similar in this dynamics of VSD and pulmonary stenosis, such as in the transposition of the great arteries and in the double entry way of the LV or RV, in the atresia of the atrioventricular valves and in the corrected transposition of the great arteries. Other elements of the usual complementary exams differentiate them.

### Conduct:

In view of the balance of pulmonary and systemic flows over time, with no signs of hypoxemia and / or heart failure and in the presence of good physical tolerance, the continuity of the expectant clinical conduct was considered.

## Discussion

The natural evolution of this patient until adulthood highlights unfavorable elements, although she has been shown to be in good clinical and hemodynamic conditions. They are acquired characters that interfere with the evolution over a longer period of time. They correspond to the increase in cardiac cavities, due to accentuated pulmonary flow in the previous period of time, and to the progression of pulmonary stenosis, with cardiac hypertrophy and dilation. Despite the maintenance of good ventricular function, the patient is subject to the appearance of other adverse factors such as accentuated arrhythmias, diastolic heart failure, the appearance of progressive hypoxemia, of infective endocarditis, causes of the probable evolutionary clinical lack of control.^1^

On the other hand, little can be offered at this point, from the surgical point of view, as the presumed technique as the most appropriate would be Fontan's functional, contraindicated by the current absence of hypoxia. The corrective technique would be very difficult due to the presence of unrelated VSD and the anterior aorta.^2^ Hence, in similar cases in childhood, one wonders whether it would be more convenient to try to correct it, in that age group, even with a considerable surgical risk.

When recalling surgical techniques, subaortic VSD involves tunneling with bovine pericardium, dacron or goretex of blood flow from the LV to the aorta. This defect can be amplified when restricting the flow, on the anterior face of the same, thus avoiding the infero-dorsal conduction beam. In the presence of pulmonary stenosis, the correction is similar to that performed in the tetralogy of Fallot with resection of the infundibular muscle, through the atrial route or by right ventriculotomy, in addition to the pulmonary valvotomy with enlargement of the pulmonary ring and subsequent placement of the monocuspid. A valve graft between the RV and the pulmonary trunk may be necessary, when it is located posteriorly or when the coronary artery is positioned in the ventricular outflow tract, close to the pulmonary ring. In the absence of correction, when the RV is hypoplastic, it guides the total cavopulmonary operation as described in the single functional or anatomical ventricle. In turn, in subpulmonary VSD, the LV is directed to the pulmonary trunk. Thus, arterial and coronary artery exchange follow the same tactics recommended according to Jatene's correction.

Postoperative evolution generally follows the preferred and necessary technique depending on the anatomical type. More intense problems are seen in the postoperative management when placing connection tubes between the RV and the pulmonary trunk, in view of obstruction and/or valve failure in the evolution.

Arrhythmias can complicate the evolution when in association with atrial isomerism, ventricular dysfunction and in postoperative residual defects.

Due to this clinical presentation, due to the specific association of pulmonary stenosis and unrelated VSD, the functional condition becomes more dependent on the repercussion of the obstructive lesion. Pulmonary stenosis can decrease the repercussion of VSD and there is a counterbalance such that the pulmonary and systemic flows are equivalent. Therefore, the patient can remain without volume overloads and symptoms and progress properly until adulthood, without manifestation. However, systolic overload of the RV due to pulmonary stenosis and the slight repercussion of left ventricular volume can, in the long run, cause evolutionary problems such as heart failure, arrhythmias, which obscure the results, and put life at risk.^1^

In the presence of unrelated VSD as in the case on display, the technique devised by Barbero-Marcial^3^ directs the LV to the aorta with tunneling with patches from the VSD to the aortic valve, and with relief of pulmonary stenosis, applied with relative success in survival rate of 86.5% after 10 years.^4^^,^^5^ Another technique, such as directing VSD to the pulmonary artery and subsequent arterial exchange is also feasible.

It is concluded, therefore, that the most appropriate management in these patients, even with balance of flows, systemic and pulmonary, is that of corrective intervention at earlier ages, even if the patient is in good clinical condition.^5^^–^^7^
